# Desafios da pandemia de coronavírus para os intensivistas brasileiros: presente e futuro

**DOI:** 10.5935/0103-507X.20210052

**Published:** 2021

**Authors:** Suzana Margareth Lobo, Patrícia M. Veiga de Carvalho Mello

**Affiliations:** 1 Divisão de Terapia Intensiva, Hospital de Base, Faculdade de Medicina de São José do Rio Preto - São José do Rio Preto (SP), Brasil.; 2 Divisão de Terapia Intensiva, Hospital de Terapia Intensiva. Centro Universitário UniFacid - Teresina (PI), Brasil.

## Comments

The first cases of severe acute respiratory syndrome coronavirus 2 (SARS-COV-2) in Brazil was reported in February 2020. Despite the extensive reach of the Brazilian public health system and its impressive capillarity capacity throughout the entire country, not a single county or community was spared from coronavirus disease 2019 (COVID-19). The mitigation phase of the pandemic was initially effective in flattening the curves of cases and hospitalizations in many regions, with a few exceptions. However, during the second wave, the number of cases surpassed more than 14 million, and Brazil has now recorded nearly half a million deaths. To prepare for future disasters, we need to understand what has happened in our country and how can we do better.

The lack of agreement and coordination within federal, state and municipal governments led to a leadership crisis while the virus was spreading at its highest rate. In the span of one year, we have had four ministers of health. The crisis was characterized by miscommunication to the public, the lack of an effective testing policy, insufficient lockdowns in places with an imminent health system collapse, communities manifesting disbelief in the severity of the disease and the pursuit of drugs with unproven efficacy. The economy and health were dichotomized as if they were mutually exclusive. Social distancing was not an option for informal workers, and low-income families who received emergency cash assistance did not have their needs met. An unhelpful social media battle over the virus and all aspects of the response has raged throughout the duration of the pandemic; moreover, despite the extensive capacity of our national immunization program, priority was not given to a vaccine acquisition strategy.

The P1 variant of SARS-CoV-2 originated in Manaus at the end of 2020, and it placed the entire country on a red alert level due to this variant's higher transmissibility and reinfection ability.^(1)^ It led to internationally known scenes of mass grave burials and oxygen shortage crises. This created a need to initiate interstate transfers of patients who were infected with the new P1 variant. Within a couple of weeks, simultaneous health system collapses in most states were taking place. By the end of March 2021, intensive care unit (ICU) bed occupancy was greater than 90% in most states. A lack of oxygen, medications, equipment, and health care professionals compromised care not only for COVID-19 patients but also for those who needed care for other medical conditions.

On a single day in March 2021, Brazil recorded more than 100,000 new cases of the disease. In April, more than 4,000 deaths were recorded in one day. In addition, standardized mortality rates for ICU patients increased to 1.18 for the first time after being below 1.0 for 3 years according to the Brazilian ICU Register.^(2)^

Brazil complied with the World Health Organization's (WHO) recommendation regarding the number of ICU beds per inhabitant, which is one to three beds per 10,000 inhabitants. Nonetheless, most of these beds were offered in the private health system and were largely located in more developed areas of the country, leaving major resource deficits in the northern and northeastern regions.^(3)^ For more than 80% of our citizens who depend on the public health care system, there were major deficits of ICU beds and in access to high-quality care.

In the last 12 months, ICU bed availability increased by approximately 150%, going from 11,300 to 28,100.^(4)^ Despite this impressive surge capacity, the scarcity of qualified staffing limited the achievement of better results. Caregiver burnout among frontline staff was not only extremely common but also prolonged. In a survey among 2,000 health care professionals from the front line, signs of burnout were perceived by 90% of the respondents in June 2020 and 95% in March 2021. The rapid and excessive increase in bed capacity overwhelmed the entire system and profoundly compromised patient outcomes. Thus, an ICU bed is much more than a bed and equipment, and the lack of specialized human resources contributed to the high lethality of COVID-19, particularly within the most vulnerable populations and in the regions with lower capacities of qualified staff.^(5-7)^

We estimated that 21,200 physicians of other specialties joined the ICUs to care for COVID-19 patients, with few or no intensivists in charge. The establishment of teams in "two levels" of care would increase the capacity of intensive care professionals to supervise the care of an increasing number of patients. A document proposing such a model was sent by *Associação de Medicina Intensiva Brasileira* (AMIB) to the Ministry of Health and the governors. However, this model was implemented in very few new ICUs.

As we struggle to vaccinate our population, we are afraid of a third wave beginning within an already overwhelmed health system. We are now in a phase of chronic contingency or chronic crisis, depending on the region. Recovery may require years.

As of now, we believe that intensive care medicine has finally had its importance recognized in our country. It is no longer a "hidden specialty", and its professionals have finally been recognized and valued in our country. Our scientific community has also achieved international recognition. However, the list of future challenges is long. We must be ready to care for patients suffering from post-COVID-19 long-term conditions and other non-COVID patients, as well as be prepared for future pandemics. A strong preparedness plan for catastrophes integrating actions from prehospital to complex hospital care must be in place. Strong programs for training specialized critical care teams are urgently needed, so that critical care becomes a more attractive career path for these professionals. Moreover, equity in access to critical care must be commonplace.

Finally, the medical response to a sanitary crisis must involve each and every part of the nation, with each part coordinating its sphere of responsibility in concert with the overall strategy. Medical decisions must be driven by science. The COVID-19 crisis will end when each citizen finally understands that the actions required to bring an end to the pandemic are not actions that occur inside our nation's hospitals but rather the everyday actions that citizens perform in their daily lives.
